# Extranodal Natural Killer/T-cell Lymphoma With Skin Involvement: A Case Report

**DOI:** 10.7759/cureus.100695

**Published:** 2026-01-03

**Authors:** Evelyn Itzamara Figueroa Saavedra, María Fernanda Molina Hernández, Mario Shuchleib Cukiert, Cristina Berumen-Glinz, Marcela Hernández-Vera, Judith Dominguez-Cherit, Sonia Toussaint Caire

**Affiliations:** 1 Dermatology, "Dr. Manuel Gea González" General Hospital, Mexico City, MEX; 2 Medicine, "Dr. Manuel Gea González" General Hospital, Mexico City, MEX; 3 Dermatopathology, "Dr. Manuel Gea González" General Hospital, Mexico City, MEX

**Keywords:** angiocentricity, cd56, chemotherapy, epstein-barr virus, radiotherapy

## Abstract

Extranodal natural killer/T-cell lymphoma (ENKTL) is an aggressive neoplasm derived from cytotoxic T cells or natural killer (NK) cells, with higher prevalence in Asia and Latin America. Epstein-Barr virus (EBV) infection plays a crucial role in its pathogenesis, being detected in most cases through in situ hybridization for EBV-encoded RNA (EBER). ENKTL predominantly affects men between 40 and 50 years of age, mainly involving the upper aerodigestive tract, particularly the nose and nasopharynx. However, it can also present in extranasal sites, where it tends to behave more aggressively. Clinical manifestations include cutaneous lesions with variable presentations that often mimic infections, delaying diagnosis. In limited-stage disease, radiotherapy (RT) is the treatment of choice, while advanced disease requires chemotherapy regimens, although no universally accepted standard protocol currently exists.

## Introduction

Extranodal natural killer/T-cell lymphoma (ENKTL) is a rare and aggressive subtype of non-Hodgkin lymphoma derived from T cells and natural killer (NK) cells that arises outside lymph nodes. It accounts for approximately 3%-10% of all non-Hodgkin lymphomas, with a higher incidence in Asia and Latin America [1]. Epstein-Barr virus (EBV) infection plays a central role in malignant transformation.

More than 80% of ENKTL cases involve the nasal cavity, nasopharynx, oropharynx, Waldeyer’s ring, and other sites of the upper aerodigestive tract, while approximately 20% arise in non-nasal locations such as the skin, testicles, gastrointestinal tract, muscles, and salivary glands [2,3]. Cutaneous manifestations may mimic infectious processes, leading to diagnostic delays. Diagnosis requires an integrated approach that includes histopathology, immunophenotyping, EBV detection, T-cell receptor gene rearrangement analysis, and imaging studies, particularly positron emission tomography/computed tomography (PET/CT). Plasma EBV viral load serves as a prognostic and disease-monitoring marker. Risk stratification models such as prognostic index of natural killer cell lymphoma (PINK), PINK-EBV-extended version (PINK-E), and Korean prognostic index (KPI) guide therapeutic decision-making [4,5].

Cutaneous involvement in ENKTL is relatively uncommon but clinically significant, accounting for approximately 10%-20% of non-nasal cases. Skin lesions frequently resemble bacterial or inflammatory soft tissue infections, representing a major diagnostic challenge. Importantly, cutaneous disease has been associated with a more aggressive clinical course and poorer prognosis. In addition, hemophagocytic lymphohistiocytosis (HLH) is a recognized and severe complication of ENKTL, reported in approximately 7%-13% of patients, and is associated with high mortality, particularly in advanced or extranodal disease [2,6].

We report the case of a 30-year-old woman with ENKTL presenting with primary cutaneous involvement of the right lower extremity, initially misdiagnosed as a soft tissue infection, who subsequently developed HLH. This case highlights the clinical relevance, diagnostic complexity, and prognostic implications of cutaneous presentations of ENKTL in young patients.

## Case presentation

A 30-year-old woman originally from and residing in Mexico City, employed as a cleaning worker, with no relevant past medical history, presented with a lesion on the right lateral malleolus that appeared in September 2024 as a small “pimple” and rapidly progressed into three ulcers. She received multiple courses of antibiotic therapy, including levofloxacin, metronidazole, ceftriaxone, and clindamycin, due to suspicion of a soft tissue infection, without clinical improvement. Over the following weeks, she developed fever, night sweats, anorexia, early satiety, and an unintentional weight loss of 10 kg over two months.

**Figure 1:**
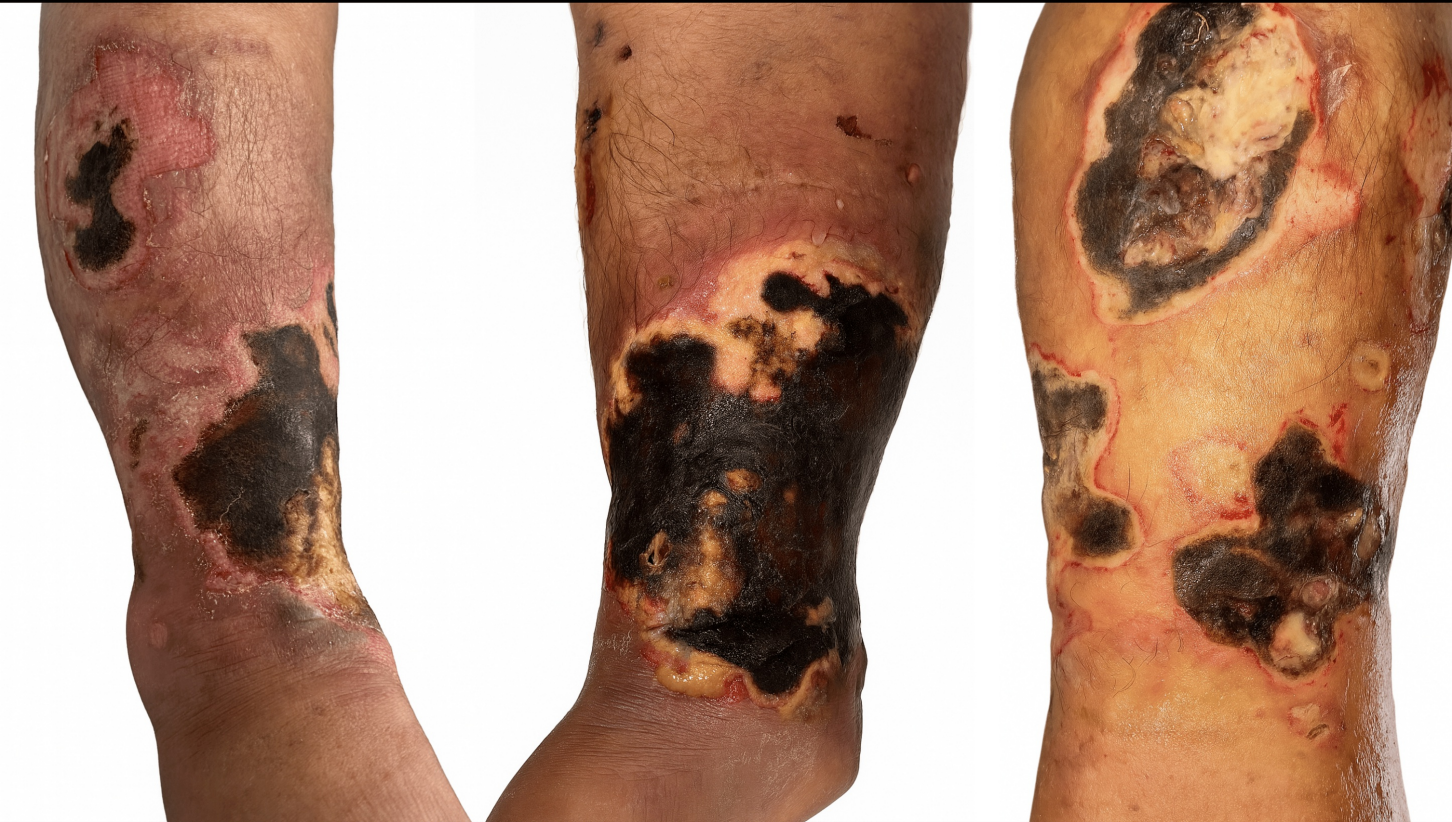
Dermatosis of the right lower extremity Dermatosis of the right lower extremity involving the anterior, posterior, and lateral aspects of the leg, characterized by three ulcers of variable size (6-23 cm in diameter), with irregular but well-defined borders and wound beds containing fibrin, eschar, and variable amounts of slough.

In January 2025, she was admitted to the Emergency Department after a fall from standing height associated with hypotension. Physical examination revealed dermatosis localized to the right lower limb, involving the anterior, posterior, and lateral aspects of the leg. Three ulcers measuring between 6 and 23 cm in diameter were observed, with irregular but well-defined borders and wound beds containing fibrin, slough, and variable amounts of necrotic eschar (Figure 1).

**Figure 2:**
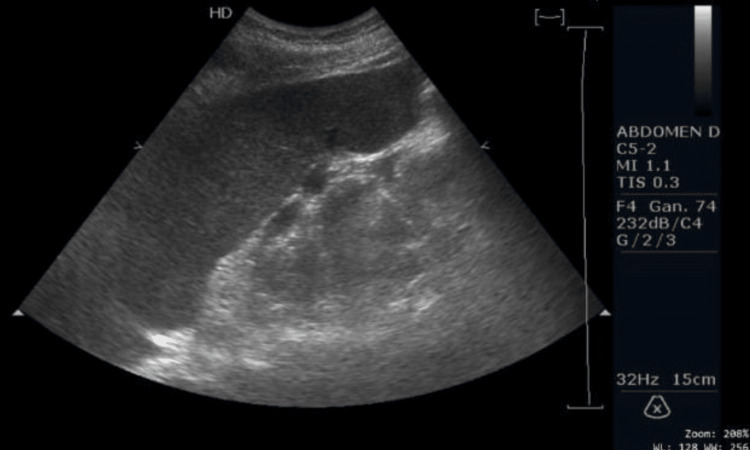
Abdominal ultrasonography Spleen in normal position and orientation measures 15.5 cm × 6.21 cm × 8.98 cm (splenomegaly), with a volume of 452 cm³.

As part of the diagnostic work-up, wound cultures, laboratory studies, and skin biopsies were obtained. Empirical antibiotic therapy with metronidazole and ceftriaxone was initiated. Initial laboratory tests demonstrated leukopenia, anemia, thrombocytopenia, and elevated lactate dehydrogenase levels (Table 1). Abdominal ultrasonography revealed splenomegaly (Figure 2).

**Table 1:** Laboratory test

Laboratory Test	Result	Reference Range
Leukocytes	1,400 µL	4,000-12,000 µL
Hemoglobin	10.87 g/dL	10.90-14.30 g/dL
Platelets	40,000 µL	179,000-408,000 µL
Lactate dehydrogenase	752 U/L	140-271 U/L

**Figure 3:**
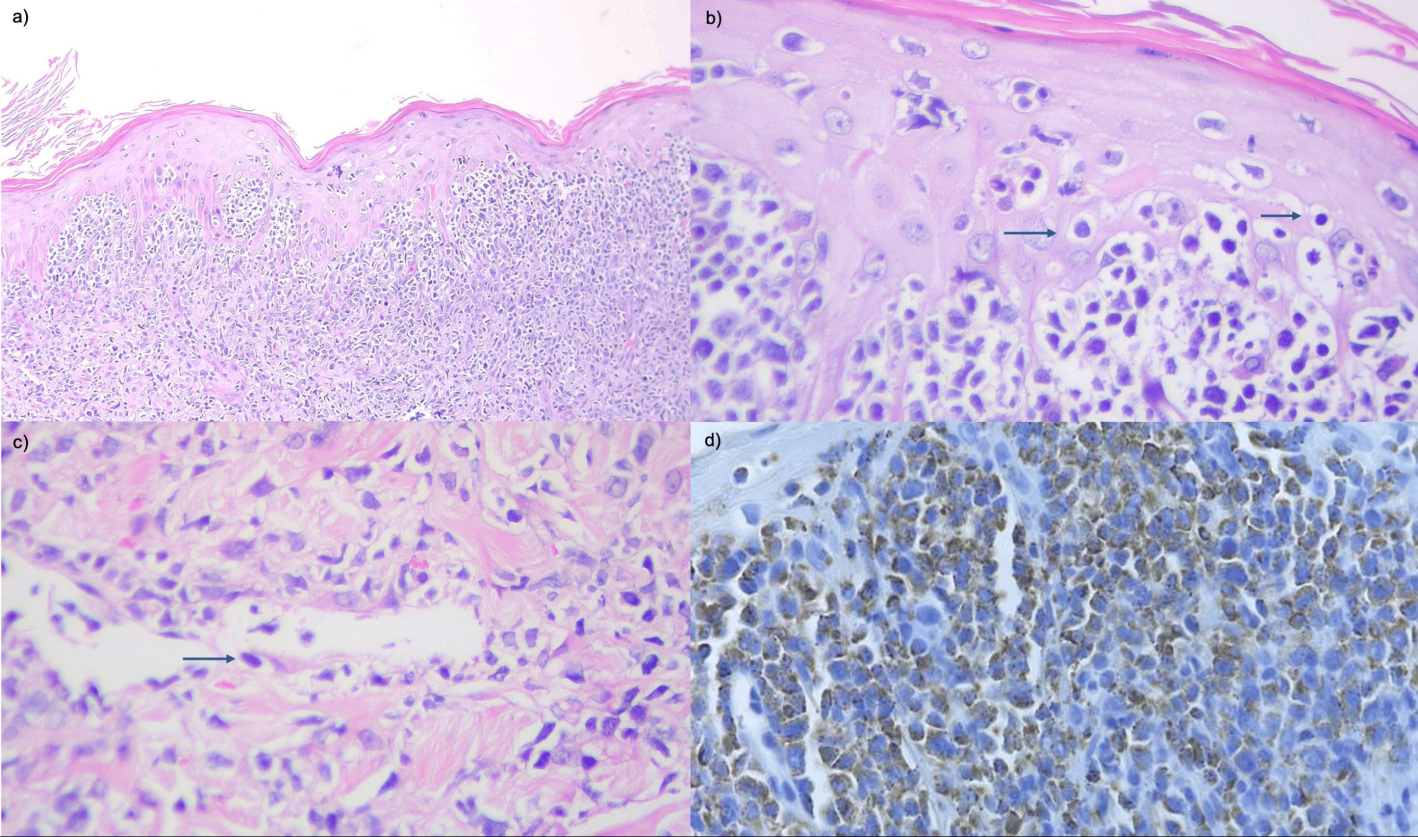
Histopathological examination a) Dense atypical lymphoid infiltrate involving the papillary and superficial reticular dermis. b) Epidermotropism characterized by infiltration of atypical lymphoid cells within the superficial epidermal layers (arrows). c) The infiltrate displays a perivascular and periadnexal distribution, with evidence of angioinvasion and angiodestruction, in selected vessels (arrow). d) Cytoplasmic TIA-1 immunostaining positive in neoplastic lymphocytes.

Wound cultures grew *Enterococcus faecalis* and extended-spectrum β-lactamase (ESBL)-producing *Escherichia coli*, prompting a change in antimicrobial therapy to meropenem and ampicillin. Histopathological examination of the ulcer biopsy revealed an atypical lymphoid infiltrate involving the dermis and subcutaneous tissue, with epidermotropism and angioinvasion by lymphocytes positive for CD3, CD30, CD56, TIA-1, and EBV-encoded RNA (EBER), confirming the diagnosis of ENKTL with cutaneous involvement (Figure 3).

Subsequently, laboratory findings were consistent with HLH, including hyperferritinemia, hypofibrinogenemia, hypertriglyceridemia, and fever >38.5 ºC (Table 2). The calculated HScore was 190 points, indicating a high probability of hemophagocytic syndrome. The hematology service initiated treatment with dexamethasone at a dose of 10 mg/m² (16 mg every 24 hours) and intravenous fibrinogen replacement (3.5 g).

**Table 2:** Laboratory test

Laboratory Test	Result	Reference Range
Serum ferritin	2,525 ng/mL	11-306 ng/mL
Fibrinogen	67 mg/dL	238-498 mg/dL
Triglycerides	881 mg/dL	40-150 mg/dL

During hospitalization, the patient developed hospital-acquired pneumonia with progressive respiratory deterioration, requiring admission to the intensive care unit for advanced airway management. Despite supportive care and pharmacologic treatment, she died due to refractory respiratory failure.

## Discussion

According to the 5th edition of the WHO Classification of Haematolymphoid Tumors (WHO-HAEM5), this entity is termed “extranodal NK/T-cell lymphoma” because it may involve multiple extranodal sites; therefore, the designation “nasal type” has been removed, as it no longer reflects a specific anatomical location [4].

This lymphoma typically affects patients between 40 and 50 years of age, with a male-to-female ratio of 2-3:1. Approximately 70%-90% of cases are diagnosed at Ann Arbor stage I or II [1]. EBV plays a pivotal role in ENKTL pathogenesis. EBER is detected in most cases by in situ hybridization and serves as both a critical diagnostic and prognostic biomarker [2].

Cutaneous lesions associated with ENKTL exhibit wide clinical variability, including subcutaneous nodules, abscesses, ulcers, erythematous plaques with necrosis, purpuric plaques, and edema. The lower extremities represent the most frequent anatomical location. These manifestations may mimic cellulitis, abscesses, or infectious panniculitis, as occurred in our patient, who was repeatedly treated as having a soft tissue infection [3,7].

Diagnosis requires a multidisciplinary approach integrating histopathology, immunophenotyping, molecular studies, and imaging. Histopathological examination typically reveals a diffuse or perivascular dermal lymphoid infiltrate, often extending into the subcutis. Epidermotropism, interface dermatitis, pseudoepitheliomatous hyperplasia, and necrosis may be observed. Cytological features range from small to large atypical lymphocytes, with medium-sized cells being the most common. Angiocentric growth is observed in 40%-70% of cases, with vascular involvement frequently demonstrating fibrinoid change and erythrocyte extravasation [8].

Approximately 7% of patients develop secondary HLH. Bone marrow involvement and B symptoms are observed in approximately 10% and 35% of patients, respectively. Furthermore, systemic dissemination is present in up to 40% of cases at the time of diagnosis [7].

The classic immunophenotype resembles that of normal NK cells, characterized by expression of CD45, CD2, and CD56. Surface CD3 and T-cell receptor expression are typically absent, although cytoplasmic CD3ε may be positive. Lack of CD4, CD5, CD8, and CD20 expression is characteristic, although up to 20% of cases may express CD8 [9]. Tumor cells demonstrate a cytotoxic phenotype with expression of perforin, granzyme B, TIA-1, CD2, and CD3ε. Detection of EBER is essential for diagnosis according to WHO criteria; its absence effectively excludes ENKTL [10].

The principal differential diagnoses include subcutaneous panniculitis-like T-cell lymphoma (SPTCL) and γδ T-cell lymphoma (GDTCL) [10]. The WHO diagnostic criteria include essential and desirable features, which are summarized in Table 3 [11].

**Table 3:** WHO classification criteria for extranodal natural killer/T-cell lymphoma Source: [11] EBER, EBV-encoded RNA; EBV, Epstein-Barr virus

Essential criteria	
Dermal and/or subcutaneous infiltration by lymphoma cells with variable morphology	
Cytotoxic natural killer-cell or T-cell phenotype	
EBER positivity in the majority of lymphoma cells	
Desirable criteria	
Angiocentric growth and necrosis	

Imaging plays a crucial role in diagnosis and staging. PET/CT is the current standard, providing an accurate assessment of disease extent and treatment response [12]. Bone marrow biopsy remains indispensable for evaluating marrow involvement and carries important prognostic implications [13].

Prognosis is guided by several validated risk models, including the PINK, its PINK-E, the KPI, and the Nomogram-Based Revised Risk Index (NRI) [14].

For early-stage ENKTL, radiotherapy (RT) is the cornerstone of treatment, with reported five-year survival rates approaching 70%. For Ann Arbor stage I/II disease, doses ≥50 Gy are recommended; intensive concurrent regimens may use 40-54 Gy. Low-risk patients may achieve excellent outcomes with RT alone (five-year overall survival 88.8% and progression-free survival 79.2%). Intermediate- and high-risk patients benefit from combined chemoradiotherapy, as systemic relapse rates reach 25%-40% with RT alone [15].

Because ENKTL commonly expresses P-glycoprotein (MDR1), chemotherapeutic agents susceptible to this resistance mechanism are avoided. Instead, L-asparaginase-based or platinum-based regimens are preferred. The SMILE regimen (dexamethasone, methotrexate, ifosfamide, L-asparaginase, and etoposide) combined with RT achieves complete remission rates of up to 82%, although it is associated with significant hematologic toxicity [16].

Advanced disease (Ann Arbor stage III/IV) or refractory disease, observed in 20%-30% of patients, is treated with L-asparaginase-based chemotherapy, with consolidative RT when feasible. Hematopoietic stem cell transplantation is reserved for selected high-risk patients who fail to achieve a second complete remission [16].

ENKTL frequently overexpresses programmed death-ligand 1 (PD-L1), mediated by EBV latent membrane protein 1 (LMP1) through activation of nuclear Factor kappa-light-chain-enhancer of activated B cells (NF-κB) and mitogen-activated protein kinase (MAPK) pathways, contributing to immune evasion [17]. Immune checkpoint inhibitors targeting PD-1/PD-L1 have demonstrated promising activity in retrospective and early-phase studies, particularly in L-asparaginase-refractory patients [18,19]. Agents such as pembrolizumab, sintilimab, and daratumumab have shown encouraging efficacy and tolerability, although further prospective studies are required [20].

CD30 is expressed in approximately 50% of cases. Brentuximab vedotin, alone or in combination with bendamustine, has induced complete remissions in refractory disease. CD38, frequently expressed and associated with poor prognosis, has been effectively targeted with daratumumab. Finally, aberrant JAK3-STAT (Janus kinase 3-signal transducer and activator of transcription) signaling in ENKTL supports ongoing investigation of JAK inhibitors such as ruxolitinib [9].

Poor prognostic factors include multiple cutaneous lesions, lower extremity involvement, presence of B symptoms, and elevated circulating EBV DNA levels. Reported overall survival ranges from 12 to 29 months, with five-year survival rates between 22% and 32% [7].

In the present case, the patient exhibited several adverse prognostic factors, including multiple ulcers of the right lower extremity, involvement of the lower limb as the primary site, and prolonged B symptoms preceding hospitalization.

## Conclusions

ENKTL is a rare but highly aggressive lymphoid neoplasm with higher prevalence in Asia and Latin America. The presence of rapidly evolving ulcerated cutaneous lesions, accompanied by systemic symptoms and early laboratory abnormalities, should raise clinical suspicion of ENKTL and prompt a timely biopsy. Therapeutic advances, particularly L-asparaginase-based chemotherapy regimens, have contributed to improved patient outcomes. Nevertheless, the aggressive nature of this disease requires early diagnosis and a multidisciplinary therapeutic approach, as timely intervention may significantly alter the poor prognosis that characterizes this entity.
